# Efficacy and safety of sorafenib for advanced renal cell carcinoma: real-world data of patients with renal impairment

**DOI:** 10.18632/oncotarget.24779

**Published:** 2018-04-10

**Authors:** Katsunori Tatsugami, Mototsugu Oya, Koki Kabu, Hideyuki Akaza

**Affiliations:** ^1^ Department of Urology, Graduate School of Medical Science, Kyushu University, Higashi-ku, Fukuoka City 812-8582, Japan; ^2^ Department of Urology, Keio University School of Medicine, Shinjuku-ku, Tokyo 160-8582, Japan; ^3^ Medical Affairs Oncology and Hematology, Bayer Yakuhin, Ltd., Chiyoda-ku, Tokyo 100-8265, Japan; ^4^ Department of Strategic Investigation on Comprehensive Cancer Network, Interfaculty Initiative in Information Studies/Graduate School of Interdisciplinary Information Studies, The University of Tokyo, Meguro-ku, Tokyo 153-8904, Japan

**Keywords:** sorafenib, advanced RCC, eGFR, real-world data, propensity score matching

## Abstract

**Background:**

We retrospectively analysed the efficacy and safety of sorafenib in patients with advanced renal cell carcinoma with renal impairment.

**Methods:**

Patients were divided into two groups by an estimated glomerular filtration rate (eGFR) cut-off of 45 mL/min/1.73 m^2^. Background factors considered to affect prognosis were well balanced by propensity score matching between the groups. Demographics, dose modification, adverse events, tumour response, progression-free survival, and renal function (eGFR) were evaluated.

**Results:**

Among 935 and 2008 patients with an eGFR of <45 and ≥45, respectively, 613 pairs were matched. The mean starting dose was significantly lower in patients with an eGFR of <45; however, the mean daily dose, median treatment duration, progression-free survival, and tumour response were similar between the groups. In terms of safety, no significant differences were found in serious adverse events, although cytopaenia (16.6% vs 10.6%) and renal dysfunction (4.4% vs 0.7%) were higher in patients with an eGFR of <45 than ≥45 in all adverse events. There were also no differences in dose modification, including dose reduction, dose interruption, and treatment discontinuation.

**Conclusion:**

Throughout the 12-month observation period, sorafenib in patients with an eGFR of <45 and ≥45 showed similar safety and efficacy, and treatment was continued without affecting renal function.

## INTRODUCTION

Sorafenib, a vascular endothelial growth factor receptor–tyrosine kinase inhibitor (VEGFR-TKI), was approved as a first-in-class molecular-targeted drug for patients with unresectable and recurrent renal cell carcinoma (RCC) due to the extension of progression-free survival (PFS) in a phase 3 study [1]. VEGFR is highly expressed on vascular endothelial cells and glomerular epithelial cells (podocytes). This receptor regulates the formation of fenestrations and slit diaphragms in vascular endothelial cells and podocytes, respectively, and blockade of VEGFR signal transduction leads to proteinuria [2, 3]. Therefore, although tumour shrinkage is expected in patients with RCC treated with VEGFR-TKIs, there is concern that such treatment may induce a decrease in renal function. Because a limited number of reports have described the relationship between VEGFR-TKIs and renal function in patients with RCC, studies on the safety and efficacy of VEGFR-TKIs for patients with RCC exhibiting low renal function are needed.

The kidney is a multifunctional organ that plays an important role in maintaining bodily homeostasis by excreting body wastes, regulating haematopoiesis, and balancing the electrolytes in the body. Chronic kidney disease (CKD) is generally defined by the estimated glomerular filtration rate (eGFR), an index of renal function, and is considered to be present when the eGFR is <60 mL/min/1.73 m^2^ for >3 months. CKD increases the risk of end-stage renal disease and death due to cardiovascular disease (CVD) [4]. In particular, studies have shown that when the eGFR decreases to <45 mL/min/1.73 m^2^, the all-cause mortality, CVD events, and hospitalisation rates rapidly increase [5, 6]. In the present study, we analysed the safety and efficacy of sorafenib in patients with advanced RCC with an eGFR of <45 mL/min/1.73 m^2^ (CKD stage <G3b) using real-world data by propensity score matching.

## RESULTS

### Patients’ demographics according to eGFR

Before matching, patients with an eGFR of <45 *vs* ≥45 mL/min/1.73 m^2^ showed significant differences in age (69.2 ± 9.0 *vs* 63.9 ± 10.8 years, *P* < 0.0001), eGFR (33.3 ± 11.0 *vs* 63.9 ± 17.7 mL/min/1.73 m^2^, *P* < 0.0001), prior surgery (85.7% *vs* 81.5%, *P* = 0.0055), metastasis (bone) (27.5% *vs* 33.7%, *P* = 0.0008), and favourable/intermediate/poor MSKCC risk (14.0/66.7/5.6% *vs* 20.2/59.7/6.2%, *P* = 0.0008). However, the patients’ background factors were balanced after matching except for metastasis to the contralateral kidney (9.0% *vs* 5.9%, *P* = 0.0384) (Table 1).

**Table 1:** Patients’ baseline demographics

	Before matching			After matching		
Variables	eGFR of <45	eGFR of ≥45	*P*-value	eGFR of <45	eGFR of ≥45	*P*-value
	(*n* = 935)	(*n* = 2008)		(*n* = 613)	(*n* = 613)	
Sex						
Male/Female	721 (77.1)/214 (22.9)	1499 (74.7)/509 (25.4)	0.1488	477 (77.8)/136 (22.2)	461 (75.2)/152 (24.8)	0.2811
Age, y	69.2 ± 9.0	63.9 ± 10.8	<0.0001	68.9 ± 8.5	69.0 ± 8.3	0.7234
Weight, kg	58.8 ± 10.3	59.2 ± 12.0	0.4137	58.9 ± 10.3	58.2 ± 11.2	0.2937
BMI, kg/m^2^	22.4 ± 3.2	22.3 ± 3.6	0.8872	22.4 ± 3.1	22.4 ± 3.5	0.9257
Mean eGFR, mL/min/1.73 m2	33.3 ± 11.0	63.9 ± 17.7	<0.0001	33.8 ± 10.8	59.9 ± 14.4	<0.0001
ECOG PS			0.0918			0.4702
0	580 (62.0)	1296 (64.5)		382 (62.3)	390 (63.6)	
1	314 (33.6)	602 (30.0)		206 (33.6)	191 (31.2)	
≥2	41 (4.4)	110 (5.5)		25 (4.1)	32 (5.2)	
TNM stage			0.2917			0.6744
I	8 (0.9)	8 (0.4)		1 (0.2)	0 (0.0)	
II	4 (0.4)	5 (0.3)		1 (0.2)	2 (0.3)	
III	11 (1.2)	31 (1.5)		3 (0.5)	2 (0.3)	
IV	910 (97.3)	1962 (97.7)		608 (99.2)	609 (99.4)	
Unknown	2 (0.2)	2 (0.1)		0 (0.0)	0 (0.0)	
Prior surgery, yes/no	801 (85.7)/134 (14.3)	1637 (81.5)/371 (18.5)	0.0055	577 (94.1)/36 (5.9)	581 (94.8)/32 (5.2)	0.6177
Prior systemic anticancer therapy			0.6676			0.1459
IFN-α	689 (73.7)	1483 (73.9)	0.9246	474 (77.3)	496 (80.9)	0.1221
IL-2	260 (27.8)	544 (27.1)	0.6849	176 (28.7)	174 (28.4)	0.8994
Others	140 (15.0)	334 (16.6)	0.2540	102 (16.6)	88 (14.4)	0.2692
Primary disease^*^			0.9306			
Unresectable/metastatic	923 (98.7)	1983 (98.8)		613 (100.0)	613 (100.0)	
RCC						
Others	0 (0.0)	0 (0.0)		0 (0.0)	0 (0.0)	
Subtype			0.0687			0.2665
Clear cell carcinoma	679 (72.6)	1457 (72.6)		528 (86.1)	541 (88.3)	
Non-clear cell carcinoma	108 (11.6)	183 (9.1)		85 (13.9)	72 (11.8)	
Metastatic site						
Any	901 (96.4)	1950 (97.1)	0.2777	604 (98.5)	606 (98.9)	0.6148
Bone	257 (27.5)	676 (33.7)	0.0008	180 (29.4)	193 (31.5)	0.4197
Brain	45 (4.8)	109 (5.4)	0.4851	31 (5.1)	32 (5.2)	0.8971
Liver	140 (15.0)	314 (15.6)	0.6423	84 (13.7)	78 (12.7)	0.6128
Lung/Lung only	652 (69.7)/239 (25.6)	1430 (71.2)/500 (24.9)	0.4105	437 (71.3)/159 (25.9)	466 (76.0)/187 (30.5)	0.0601
Kidney	76 (8.1)	136 (6.8)	0.1855	55 (9.0)	36 (5.9)	0.0384
Other (including lymph nodes)	412 (44.1)	883 (44.0)	0.9634	274 (44.7)	245 (40.0)	0.0937
Proteinuria	6 (0.6)	7 (0.4)	0.2631	4 (0.7)	3 (0.5)	0.7047
CRP, mg/dL	2.8237 ± 5.6196	3.1201 ± 4.6804	0.1775	2.6429 ± 5.8132	2.3768 ± 4.1017	0.3546
MSKCC risk (1999)^†^						
Favorable/intermediate/poor	131 (14.0)/624 (66.7)/52 (5.6)	405 (20.2)/1199 (59.7)/125 (6.2)	0.0008	95 (15.5)/433 (70.6)/19 (3.1)	126 (20.6)/412 (67.2)/22 (3.6)	0.1122
Concomitant use of cytokines						
Yes/no	33 (3.5)/902 (96.5)	87 (4.3)/1921 (95.7)	0.305	19 (3.1)/594 (96.9)	30 (4.9)/583 (95.1)	0.1088

Data are expressed as *n* (%) or mean ± standard deviation.

^*^ Including multiple choices. †Patients with any line of therapy.

Abbreviations: BMI = body mass index; CRP = C-reactive protein; ECOG PS = Eastern Cooperative Oncology Group performance status; eGFR = estimated glomerular filtration rate; IFN-α = interferon-alpha; MSKCC = Memorial Sloan Kettering Cancer Center; RCC = renal cell carcinoma; TNM = tumor, node, metastasis.

### Treatment with sorafenib

The mean starting dose of sorafenib was significantly lower in patients with an eGFR of <45 than ≥45 mL/min/1.73 m^2^ (687.1 ± 192.1 *vs* 726.3 ± 159.8 mg/day, *P* = 0.0001). However, there was no significant difference in the median [interquartile range] daily dose (484.4 [388.5] *vs* 481.0 [415.5] mg/day, *P* = 0.3181), median duration of treatment (6.11 [10.22] *vs* 6.60 [9.72] months, *P* = 0.2944), or dose modifications including dose reduction (58.2% *vs* 58.7%, *P* = 0.862), dose interruption (43.9% *vs* 42.9%, *P* = 0.7295), and treatment discontinuation (70.0% *vs* 69.8%, *P* = 0.9504) (Table 2). There was no difference in the numbers of patients who discontinued sorafenib treatment due to adverse events (AEs) or insufficient efficacy (Table 2).

**Table 2:** Distribution of initial dose, median dose, dose modification, and reason for treatment discontinuation

	eGFR, mL/min/1.73 m^2^		
Variables	<45	≥45	*P*-value
	(*n* = 613)	(*n* = 613)	
Mean starting dose, mg/day	687.1 ± 192.1	726.3 ± 159.8	0.0001
Median daily dose, mg/day	484.4 [388.5]	481.0 [415.5]	0.3181
Relative dose intensity, %	65.7 ± 26.5	67.2 ± 26.5	0.3197
Median duration of treatment, mo	6.11 [10.22]	6.60 [9.72]	0.2944
Dose modification			
Reduction	357 (58.2)	360 (58.7)	0.862
Interruption	269 (43.9)	263 (42.9)	0.7295
Discontinuation	429 (70.0)	428 (69.8)	0.9504
Reason for discontinuation			
Adverse events	264 (61.5)	244 (57.0)	0.1772
Insufficient effect	130 (30.3)	142 (33.2)	0.366

Data are presented as mean ± standard deviation, median [interquartile range], or *n* (%).

Abbreviations: AE = adverse event; eGFR = estimated glomerular filtration rate.

### AEs

No significant differences were found in serious AEs (53.8% *vs* 50.9%, *P* = 0.303); however, the total cytopaenia (16.6% *vs* 10.6%, *P* = 0.0021) and total renal failure/dysfunction (4.4% *vs* 0.7%, *P* < 0.0001) were significantly higher in patients with an eGFR of <45 than ≥45 mL/min/1.73 m^2^. Other AEs were similarly observed in both groups (Table 3).

**Table 3:** Most common adverse events

			eGFR, mL/min/1.73 m^2^				
Adverse event	All	Serious	<45		≥45		*P*-value
			(*n* = 613)		(*n* = 613)		(All)
			All	Serious	All	Serious	
Any	1195 (97.5)	642 (52.4)	604 (98.5)	330 (53.8)	591 (96.4)	312 (50.9)	0.018
Hand and foot skin reaction	709 (57.8)	60 (4.9)	349 (56.9)	31 (5.1)	360 (58.7)	29 (4.7)	0.5247
Hypertension	465 (37.9)	31 (2.5)	230 (37.5)	16 (2.6)	235 (38.3)	15 (2.5)	0.7685
Rash	331 (27.0)	93 (7.6)	164 (26.8)	46 (7.5)	167 (27.2)	47 (7.7)	0.847
Lipase/amylase increase	330 (26.9)	10 (0.8)	176 (28.7)	6 (1.0)	154 (25.1)	4 (0.7)	0.1566
Diarrhoea	283 (23.1)	22 (1.8)	147 (24.0)	12 (2.0)	136 (22.2)	10 (1.6)	0.4559
Alopecia	217 (17.7)	1 (0.1)	97 (15.8)	0 (0.0)	120 (19.6)	1 (0.2)	0.0852
Hepatic dysfunction	215 (17.5)	89 (7.3)	110 (17.9)	44 (7.2)	105 (17.1)	45 (7.3)	0.7073
Cytopaenia	167 (13.6)	56 (4.6)	102 (16.6)	32 (5.2)	65 (10.6)	24 (3.9)	0.0021
Decreased appetite	127 (10.4)	21 (1.7)	66 (10.8)	13 (2.1)	61 (10.0)	8 (1.3)	0.6393
Bleeding	124 (10.1)	84 (6.9)	69 (11.3)	49 (8.0)	55 (9.0)	35 (5.7)	0.1848
Mucositis	110 (9.0)	7 (0.6)	55 (9.0)	3 (0.5)	55 (9.0)	4 (0.7)	1
Hypophosphataemia	109 (8.9)	1 (0.1)	57 (9.3)	0 (0.0)	52 (8.5)	1 (0.2)	0.6159
Fever	86 (7.0)	29 (2.4)	41 (6.7)	15 (2.5)	45 (7.3)	14 (2.3)	0.6547
Dysphonia	80 (6.5)	0 (0.0)	45 (7.3)	0 (0.0)	35 (5.7)	0 (0.0)	0.2475
Renal failure/dysfunction	31 (2.5)	12 (1.0)	27 (4.4)	9 (1.5)	4 (0.7)	3 (0.5)	<0.0001
Proteinuria, *n* (%)	21 (1.7)	0	12 (2.0)	0	9 (1.5)	0	0.5090
Fatigue	17 (1.4)	2 (0.2)	6 (1.0)	0 (0.0)	11 (1.8)	2 (0.3)	0.222

Data are presented as *n* (%).

### Tumour response

**Figure 1:**
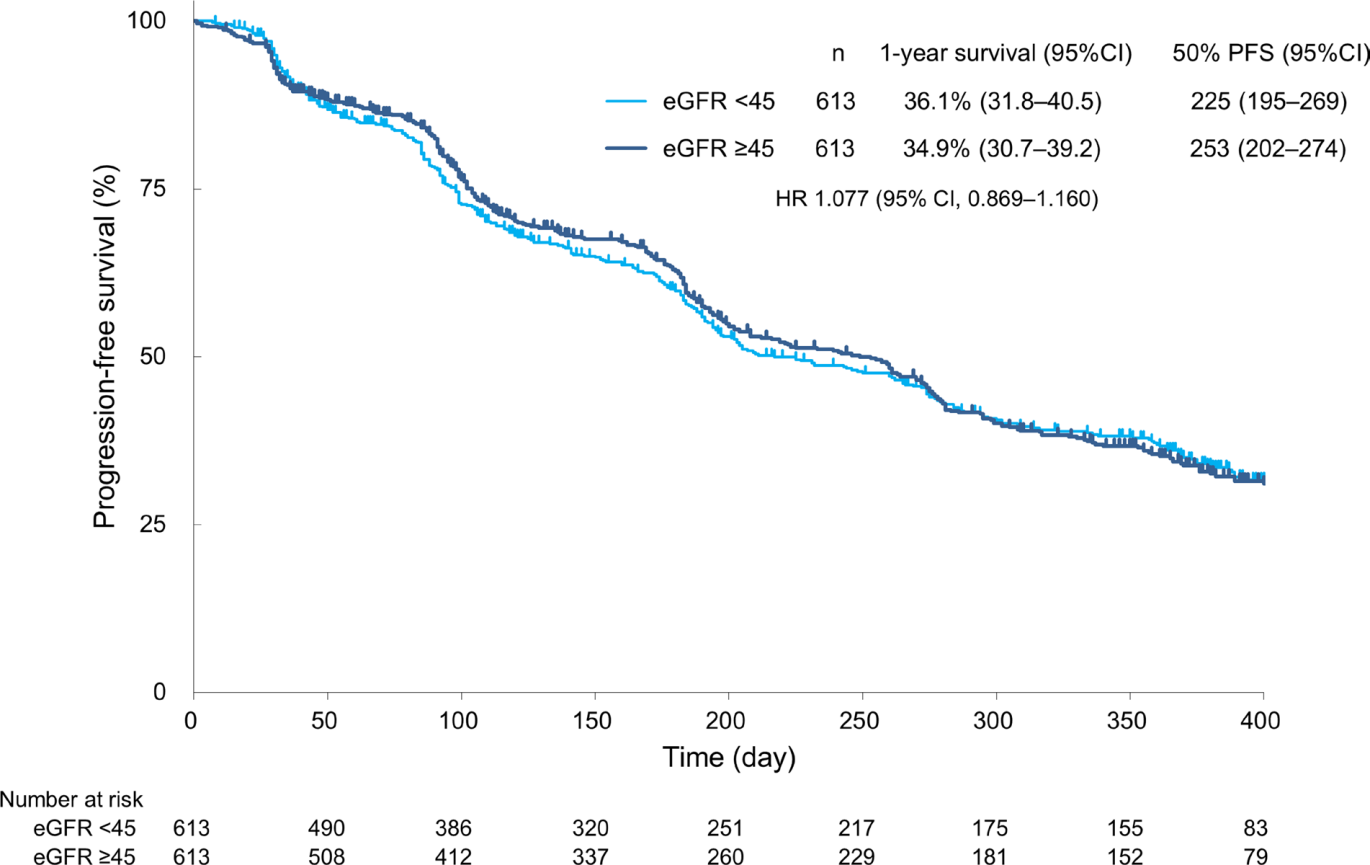
Progression-free survival PFS = progression-free survival; CI = confidence interval; HR = hazard ratio.

The rates of complete response (CR), partial response (PR), and stable disease (SD) in patients with an eGFR of <45 *vs* ≥45 mL/min/1.73 m^2^ were 1.8% *vs* 3.0%, 24.3% *vs* 26.4%, and 59.8% *vs* 57.7%, respectively. The objective response rate (CR + PR) and disease control rate (CR + PR + SD) in patients with an eGFR of <45 *vs* ≥45 mL/min/1.73 m^2^ were 26.1% *vs* 29.4% (*P* = 0.2132) and 85.8% *vs* 87.1% (*P* = 0.5350), respectively. Overall, sorafenib treatment was associated with a similar tumour response rate in both groups (Table 4). Additionally, the median PFS in patients with an eGFR of <45 *vs* ≥45 mL/min/1.73 m^2^ was 225 *vs* 253 days, respectively (hazard ratio, 1.077; 95% confidence interval, 0.869–1.160), without a significant difference (*P* = 0.9225) (Figure 1).

**Table 4:** Tumour response to sorafenib

		eGFR, mL/min/1.73 m^2^		
Variables	All	<45	≥45	*P*-value
		(*n* = 613)	(*n* = 613)	
CR, *n* (%)	27 (2.4)	10 (1.8)	17 (3.0)	0.4584
PR, *n* (%)	286 (25.3)	137 (24.3)	149 (26.4)	
SD, *n* (%)	663 (58.7)	337 (59.8)	326 (57.7)	
PD, *n* (%)	148 (13.1)	76 (13.5)	72 (12.7)	
NE, *n* (%)	5 (0.4)	4 (0.7)	1 (0.2)	
ORR, %	27.7	26.1	29.4	0.2132
DCR, %	86.5	85.8	87.1	0.5350

Abbreviations: CR = complete response; DCR = disease control rate; eGFR = estimated glomerular filtration rate; NE = not evaluable; ORR = objective response rate; PD = progressive disease; PR = partial response; SD = stable disease.

### Influence on renal function

**Figure 2:**
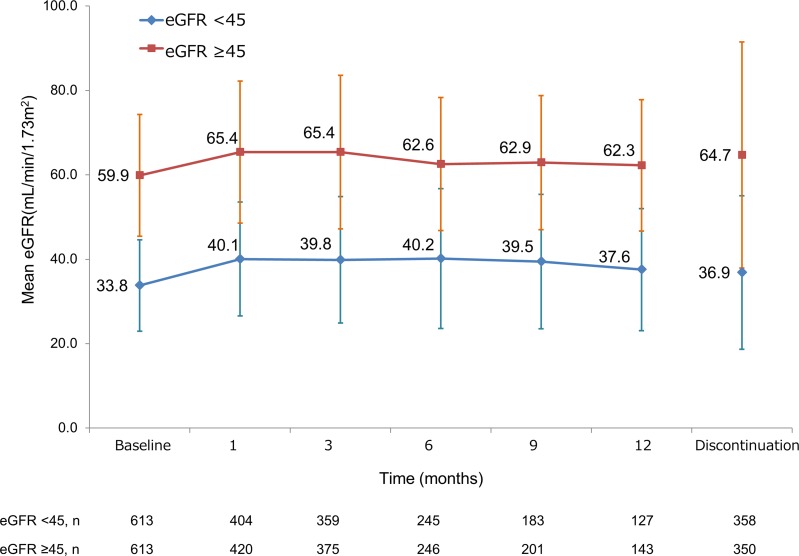
Change in renal function of overall patients over time according to eGFR eGFR = estimated glomerular filtration rate.

Because a high incidence of sorafenib-induced renal failure/dysfunction was observed in patients with an eGFR of <45 mL/min/1.73 m^2^, we analysed the impact of sorafenib on the change in renal function of patients with renal impairment between those with an eGFR of <45 and ≥45 mL/min/1.73 m^2^. The mean eGFR at baseline in patients with an eGFR of <45 *vs* ≥45 mL/min/1.73 m^2^ was 33.8 vs 55.9 mL/min/1.73 m^2^, and the renal function was retained throughout the 12-month observation period (Figure 2).

## DISCUSSION

In previous clinical studies of TKI treatment in patients with advanced RCC, an increase in creatinine or proteinuria was sometimes observed; however, such increases do not necessarily reflect clinical practice involving patients with renal impairment. For instance, in the AXIS trial, creatinine increased by 55% and 41% in patients treated with axitinib and sorafenib, respectively [7], and proteinuria was observed in 10.7% and 6.6% [8] of all patients, respectively. However, the inclusion criterion for renal function in the AXIS trial was a creatinine level of ≤1.5 mg/dL or creatinine clearance rate of ≥60 mL/min [8]; this information is not sufficient to evaluate the safety and AEs in patients with renal impairment. In one study in the clinical practice setting, patients who underwent radical nephrectomy showed a continuous decrease in renal function over time [9, 10], and most of these patients had low renal function at the time of TKI initiation. Our propensity score-matched, real-world data showed that 82.8% (2438/2943) patients had undergone nephrectomy and that 31.7% (935/2943) of patients had an eGFR of <45 mL/min/1.73 m^2^ (Table 1).

Several clinical studies to date have evaluated patients with renal impairment. In one study, 790 patients with metastatic RCC treated with sunitinib were divided into three groups by eGFR (<30, ≥60 to <30, and ≥60 mL/min/1.73 m^2^) and investigated for safety. However, accurate safety data were not reported. A low number of patients had an eGFR of <30 mL/min/1.73 m^2^ (*n* = 22), and there was no significant difference in safety or efficacy among the three groups [11]. In another study, 65 patients with metastatic RCC were treated with axitinib, and the risk factors for a decrease in renal function were found to be age (≥65 years old), baseline renal function (eGFR of <45 mL/min/1.73 m^2^), and timing of axitinib introduction (≥ third-line) [12]. Furthermore, another study showed that the risk factor for everolimus-induced acute kidney injury was pre-treatment renal dysfunction, and all patients with an eGFR of 15 to 30 mL/min/1.73 m^2^ (*n* = 2) developed acute kidney injury after everolimus treatment [13]. Like these clinical studies, the risk of TKI-induced renal impairment in the previous study was based on low pre-treatment renal function; however, the number of investigated patients with low renal function was limited. We divided 3255 patients into 2 groups using an eGFR cut-off of 45 mL/min/1.73 m^2^, which is reportedly the value at which the mortality and CVD event rates increase, and evaluated the safety of sorafenib in patients with renal impairment after propensity score matching of demographic characteristics.

With respect to the efficacy of sorafenib in patients with renal impairment, the tumour response was comparable between patients with an eGFR of <45 and ≥45 mL/min/1.73 m^2^ (Table 4, Figure 1). The reason for this result is that the median daily dose and duration of treatment were similar, and sorafenib treatment could be continued regardless of renal function despite the fact that the mean starting dose was significantly lower in patients with an eGFR of <45 mL/min/1.73 m^2^ (Table 2). It might be considered that the lower starting dose in patients with an eGFR of <45 mL/min/1.73 m^2^ was based on a concern for the safety of the patients with renal impairment. In addition, considering that treatment discontinuation due to adverse events was higher in patients with an eGFR of <45 mL/min/1.73 m^2^ (although without statistical significance) (Table 2), it is conceivable that the starting dose might have been affected by physicians’ lack of familiarity with the use of sorafenib immediately after approval by the regulatory authorities as well as physicians’ concern regarding safety in patients with poor renal function. As shown in Table 3, the number of patients with renal failure/dysfunction was higher among those with an eGFR of <45 mL/min/m^2^. When fluid loss was observed due to loss of appetite or diarrhoea by disease progression and the side effects of TKI, progression to renal function deterioration became possible. Sorafenib is mainly metabolised by the liver, and 77% of the administered drug is excreted into the faeces [14]. Therefore, the pharmacodynamics of sorafenib are likely to be comparable between patients with and without renal impairment, possibly explaining why the tumour response (Table 4), PFS (Figure 1), and AEs (Table 3) were similar in both groups based on the similar serum concentration of sorafenib. However, further study is needed in this regard.

TKI-induced renal impairment may occur by several mechanisms. The first is glomerular obstruction by inhibition of the VEGF signalling pathway. Urine filtration is mediated through the glomerular filtration barrier, which consists of podocytes, the glomerular basement membrane, and endothelial cells [15]. VEGF-producing podocytes contribute to maintenance of glomerular function via both the podocytes themselves and endothelial cells, and the inhibition of VEGF leads to collapse of the glomerular filtration barrier, resulting in proteinuria [3]. The second mechanism is glomerular deconstruction, termed thrombotic microangiopathy. Direct endothelial dysfunction is induced by VEGF inhibition, mesangiolysis, swelling of endothelial cells and schistocytes, and thrombosis [15]. The third mechanism is based on the inhibition of the production of vasodilator such as nitric oxide and prostaglandin I2 from endothelial cells, leading to hypertension [16]. Each TKI has a possibility of causing renal dysfunction, although there are some differences in their inhibitory specificities to VEGF. In the present study, sorafenib-induced renal dysfunction was observed in 31 (2.5%) patients (27 [4.4%] *vs* 4 [0.7%] among those with an eGFR of <45 *vs* ≥45 mL/min/1.73 m^2^, respectively; *P* < 0.0001). Generally, renal function tends to decrease at a higher rate in patients with lower baseline renal function [17]. At the end of the present analysis, we determined that sorafenib treatment was safely conducted for at least 12 months without a decrease in renal function in most of the patients with an eGFR of <45 mL/min/1.73 m^2^ (Figure 2). Moreover, the fact that the eGFR was similar between patients who did and did not discontinue sorafenib treatment (Figure 2) indicates that the treatment discontinuation was unlikely to be associated with the presence of renal impairment.

This study had three main limitations. The first is that it was a non-randomised retrospective study. Although propensity score matching balances patients’ demographics and apparently shows outcomes similar to those of a randomised study, certain biases cannot be denied; for example, patients with missing data regarding the matching factors at baseline were excluded from the analysis. In addition, patients’ demographics were matched using prognostic factors; however, other reported prognostic factors which were not collected at baseline could not be matched. Second, some bias might have been introduced by some physicians who may have been less familiar with the use of sorafenib because these PMS data were collected immediately after approval of sorafenib for treatment of RCC. The third limitation is the duration of the observational period, which was 12 months as required by the Pharmaceutical and Medical Devices Agency; hence, data regarding the safety and efficacy of sorafenib for >12 months could not be obtained.

In conclusion, in this study using propensity score matching, the demographics of patients with an eGFR of <45 and ≥45 mL/min/1.73 m^2^ were statistically balanced, and the safety and efficacy of sorafenib were investigated. Patients with an eGFR of <45 mL/min/1.73 m^2^ tolerated sorafenib well and showed a tumour response comparable with that of patients with an eGFR of ≥45 mL/min/1.73 m^2^ (the non-renal impairment group).

## MATERIALS AND METHODS

Study population. As reported in our earlier publications [18, 19], these data were derived from Japanese patients with histologically or cytologically confirmed unresectable or metastatic RCC who started sorafenib treatment from February 2008 to September 2009. Based on a requirement of the Pharmaceutical and Medical Devices Agency, these real-world data were prospectively collected from >3,200 patients and retrospectively analysed.

### Study design

To investigate the safety and efficacy of sorafenib for patients with RCC with an eGFR of <45 mL/min/1.73 m^2^, which reportedly increases the death rate and CVD events, the background factors affecting the patients’ prognosis were balanced by propensity score matching. Among 3255 patients, those with the following baseline data were selected for propensity score matching: age; Eastern Cooperative Oncology Group performance status; tumour, node, metastasis (TNM) classification; prior surgery; prior systemic therapy; tumour histology; metastases (liver, brain, and bone); C-reactive protein level; and 1999 Memorial Sloan Kettering Cancer Center (MSKCC) risk. In total, 2,008 patients with an eGFR of ≥45 mL/min/1.73 m^2^ and 935 patients with an eGFR of <45 mL/min/1.73 m^2^ were selected and matched with each other, resulting in 613 matching pairs (1226 patients). The patients’ demographics, dose modifications, AEs, tumour response, PFS, and renal function (as measured by eGFR) were evaluated in these two groups.

### Statistical analysis

Student’s *t*-test and the Mann–Whitney *U*-test were used to evaluate parametric and non-parametric continuous variables, respectively, and the chi-square test was used for categorical data. PFS was calculated using the Kaplan–Meier method, and statistical significance was analysed by the log-rank test unless otherwise specified. SAS version 9.1 or higher (SAS Institute Inc., Cary, NC, USA) was used for all statistical analyses.

## Abbreviations

AEs: adverse events
CKD: chronic kidney disease
CR: complete response
CVD: cardiovascular disease
eGFR: estimated glomerular filtration rate
PFS: progression-free survival
PR: partial response
RCC: renal cell carcinoma
SD: stable disease
VEGFR-TKI: vascular endothelial growth factor receptor–tyrosine kinase inhibitor
